# Rapid detection of epidermal growth factor receptor mutations with multiplex PCR and primer extension in lung cancer

**DOI:** 10.1186/1423-0127-17-37

**Published:** 2010-05-12

**Authors:** Ching-Hsiung Lin, Kun-Tu Yeh, Ya-Sian Chang, Nicholas C Hsu, Jan-Gowth Chang

**Affiliations:** 1 Department of Chest Medicine, Changhua Christian Hospital, Changhua, Taiwan; 2 Department of Pathology, Changhua Christian Hospital, Changhua, Taiwan; 3 Department of Laboratory Medicine, Kaohsiung Medical University Hospital, Kaohsiung, Taiwan; 4 Department of Veterinary Medicine, National Chung Hsiung University, Taichung, Taiwan; 5 Institute of Clinical Medicine, Kaohsiung Medical University, Kaohsiung, Taiwan; 6 Center for Excellence in Environmental Medicine, Kaohsiung Medical University, Kaohsiung, Taiwan

## Abstract

*Epidermal growth factor receptor *(*EGFR*) kinase domain mutations hyperactivate the kinase and confer kinase addiction of the non-small-cell lung cancer (NSCLC) tumor cells. Almost all of these mutations are located within exons 18-21. The -216 single nucleotide polymorphism in the promoter region is associated with increased EGFR production. We present a method for detecting these common mutations in 81 cases of NSCLC. The protocol is based on the multiplex amplification of promoter region and exons 18-21 of the *EGFR *genes in a single tube, followed by primer extension of the PCR products using various sizes of primers to detect base changes at -216 promoter region and codons 719, 746-750, 790, 858 of the *EGFR *gene. We compared the results with that from direct sequencing for detecting *EGFR *mutations in 81 cases of NSCLC. The two methods identified the same 26 mutations, but our method is superior to direct sequencing in terms of the amount of work and time required. We presented a simple and fast method to detect mutations of *EGFR *genes in NSCLC.

## Background

Lung cancer is one of the most common cancers in the world and is responsible for one third of all cancer-related death. Treatment of lung cancer mainly depends on the type of the cells that make up the cancer. Small-cell lung cancer (SCLC) which comprises about 20% of lung cancers originates from neuroendocrine cells in the bronchus. SCLC responds well to chemotherapy initially, but resistance occurs commonly. Non-small-cell lung cancer (NSCLC), comprising 80% of lung cancers, arises from lung epithelial cells, and comprises diverse histological subtypes that includes adenocarcinoma, bronchioloalveolar, squamous, anaplastic and large-cell carcinomas [1]. NSCLC is often treated with combination cytotoxic chemotherapy, but the treatment only results in a modest increase in survival. The receptor tyrosine kinases (RTKs) serve as cell signalling mediators by receptor-specific ligands. Epidermal growth factor receptor (EGFR) is a member of the ErbB family of RTKs expressed in many cases of NSCLC, and its expression is correlated with a poor prognosis [2-5]. Two EGFR small molecule inhibitors, gefitinib and erlotinib, which target the tyrosine kinase domain of EGFR have been approved for the treatment of advanced NSCLC. Females, Asians, nonsmokers, and those with bronchioloalveolar carcinoma appear to derive the most benefit from gefitinib or erlotinib [6-10]. Molecular analysis showed that the majority of responders harbored specific mutations in the gene that encodes EGFR [8,10-12]. *EGFR *kinase domain mutations occur primarily in exons 18-21 which encode part of the tyrosine kinase (TK) domain [13-15]. Besides these *EGFR *kinase domain mutations, a common single nucleotide polymorphisms (SNP) located -216 bp upstream from the initiator ATG in the promoter region also has been identified. The SNP occurs at an important binding site for the transcription factor SP1 that is necessary for activation of *EGFR *promoter activity and correlates with increased promoter activity and expression of *EGFR *mRNA [16].

In this study, we performed multiplex amplification of exons 18-21 and promoter of *EGFR *using five pair of primers followed by primer extension to detect base changes or deletions in codons 719, 746-750, 790, 858, and -216 promoter to analyze the mutational frequency in 81 cases of lung cancer, and compared the results to that obtained by direct sequencing.

## Methods

### Tissue Procurement

Tumor specimens, obtained from patients on protocols approved by the Institutional Review Board of Changhua Christian Hospital, were collected from eighty-one patients with NSCLC at the time of surgical resection before systemic treatment. All specimens were frozen immediately and stored in liquid nitrogen until DNA was extracted.

### DNA extraction, PCR and direct sequencing of the *EGFR *gene

DNA extraction was performed as previously described [17]. Five separate PCR reactions, each with the corresponding pair of primers, were used to amplify the promoter region and exons 18-21 of the *EGFR *genes (Table 1). PCR amplification of 0.2 μg DNA was performed with a denaturing step at 94°C for 5 min, then 30 sec at 94°C, 1 min at 58°C, and 1 min at 72°C for 35 cycles, followed by a final 5 min at 72°C. The PCR products were visualized on a 2.5% agarose gel. These PCR products were then subjected to direct sequencing using the same primers, and all mutations were confirmed by sequences originating from both the upstream and downstream primers. Direct sequencing was performed on a Beckman Coulter CEQ 8000 Series Genetic Analysis System (Beckman Coulter Inc., Fullerton, CA, USA) according to manufacturer instructions.

**Table 1:** PCR Primers used to amplify promoter region and exons 18, 19, 20, and 21 of the *EGFR *genes

EGFR gene	Sequence
E18-5'	5'-CTGGCACTGCTTTCCAGCAT-3'
E18-3'	5'-GCTTGCAAGGACTCTGGGCT-3'
E19-5'	5'-GCATCGCTGGTAACATCCAC-3'
E19-3'	5'-AGATGAGCAGGGTCTAGAGC-3'
E20-5'	5'-ATCGCATTCATGCGTCTTCA-3'
E20-3'	5'-AGACCGCATGTGAGGATCCT-3'
E21-5'	5'-TGACCCTGAATTCGGATGCA-3'
E21-3'	5'-ATACAGCTAGTGGGAAGGCA-3'
Promoter 5'	5'-CCTCCTCTGCTCCTCCCGAT-3'
Promoter 3'	5'-CGGGGCTAGCTCGGGACT-3'

### Multiplex PCR and primer extension analysis of mutations in EGFR-216 promoter region and exons 18, 19, 20, and 21

Multiplex PCR was used to amplify the promoter region and exons 18-21 of the *EGFR *genes in a single tube. The primers and conditions used for the multiplex PCR were the same as the PCR described above. After multiplex PCR amplification, the PCR products were purified to remove the remaining primers and unincorporated deoxynucleotide triphosphates, using the PCR-M™ Clean Up System (Viogene-biotek Co., Sunnyvale, CA, USA). After removing the primers, the products were subjected to primer extension analysis. Various concentrations of probe for -216 promoter region and exons 18-21 were added to the tube containing 1.5 μl of purified PCR products (Table 2), as well as 4 μl of ABI PRISM SNaPshot Multiplex Kit (Applied Biosystems, Foster City, CA) containing AmpliTaq^® ^DNA polymerase and fluorescently labeled dideoxynucleotide triphosphates (ddNTPs) (RGG-labeled dideoxyadenosine triphosphate, TAMRA-labeled dideoxycytidine triphosphate, ROX-labeled dideoxythymidine triphosphate, and R110-labeled dideoxyguanosine triphosphate). Each 10-μl mixture was subjected to 25 single-base extension cycles consisting of a denaturing step at 96°C for 10 sec, and primer annealing and extension at 60°C for 35 sec. After cycle extension, unincorporated fluorescent ddNTPs were incubated with 1 μl of shrimp alkaline phosphatase (SAP) (United States Biochemical Co., Cleveland, USA), at 37°C for 1 hour, followed by enzyme deactivation at 75°C for 15 min.

**Table 2:** Primer extension mutation analysis probes for -216 promoter region and codons 719, 746-750, 790, and 858 of the *EGFR *genes

Nucleotide number and sequence	Amino acid change	Sequence	Size
2155 G>T, 2155 G>A	G719C, G719S	5'-TGAATTCAAAAAGATCAAAGTGCTG-3'	25 mer

2156 G>C	G719A	5'-AAACTGAATTCAAAAAGATCAAAGTGCTGG-3'	30 mer

2235-2249 del	E746-A750 del	5'-GAAGGTGAGAAAGTTAAAATTCCCGTCGCTATCAA-3'	35 mer

2236-2250 del	E746-A750 del	5'-TCCCAGAAGGTGAGAAAGTTAAAATTCCCGTCGCTATCAAG-3'	41 mer

2237-2254 del	E746-T751 del	5'-(T)20AGTTAAAATTCCCGTCGCTATCAAGG-3'	46 mer

2240-2257 del	L747-S752 del	5'-(T)23AGTTAAAATTCCCGTCGCTATCAAGGAAT-3	52 mer

2573 T>G	L858R	5'-(T)26ACCGCAGCATGTCAAGATCACAGATTTTGGGC-3'	58 mer

2369 C>T	T790M	5'-CTCCACCGTGCAGCTCATCA-3'	20 mer

-216 G/T		5'-GGCCGCAGCAGCCTCC-3'	16 mer

The primer extension reaction products were resolved by automated capillary electrophoresis on a capillary electrophoresis platform. Briefly, 14 μl of Hi-Di™ Formamide (Applied Biosystems) and 0.28 μl of GeneScan™ -120LIZ^® ^Size Standard (Applied Biosystems) were added to 6 μl of primer extension products. All samples were loaded and run on an ABI Prism 310 DNA Genetic Analyzer (Applied Biosystems) following the manufacturer's recommendations. Following the run, samples were analyzed using GeneScan™ 3.1 application software (Applied Biosystems).

## Results

### Patients

**Figure 1:**
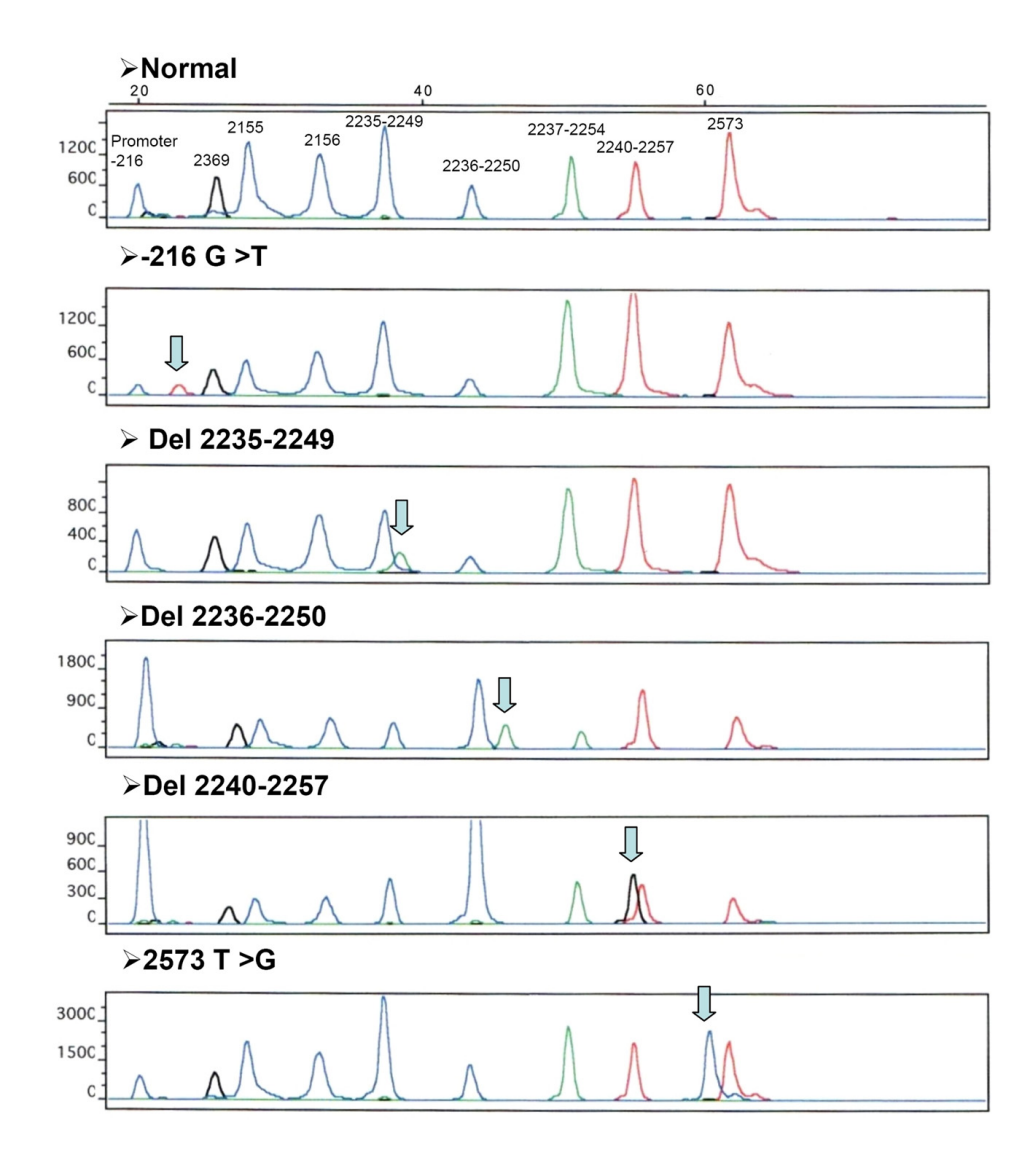
**Detection of wild-type and mutant *EGFR *by primer extension analysis**. NSCLC DNA samples of wild-type *EGFR *and ones containing the following mutations: -216 G/T, 2235-2249 del, 2236-2250 del, 2240-2257 del, and 2573 T>G.

We used multiplex PCR plus primer extension method to detect EGFR -216 promoter region and exons 18-21 mutations in 81 cases of NSCLC (Figure 1). Histologically, there were 26 adenocarcinomas, 6 bronchioloaveolar carcinomas, 33 squamous cell carcinomas, 5 adenosquamous carcinomas, and 11 other types of NSCLCs.

### Multiplex PCR and primer extension

For mutation analysis of codons 719 in exon 18, we used different-sized primers to recognize the change of the first and second base separately. In-frame deletion between codon 746 and 752 in codon 19 was analyzed with primers made to be different in size either by adding different lengths of poly(dT) tails to the 5'-end or extending the primer sequence to allow separation based on the differences in size. SNP at -216 promoter region and missense mutations which result in L858R change in exon 21 and T790M in exon 20 were each analyzed with a single probe.

The overall mutation (in EGFR TK domain and -216 promoter) rate was 32% (26 of 81).

Mutations in exons 18-21 of EGFR TK domain were detected in 19 (23%) of the 81 NSCLCs in which there were 12 adenocarcinomas, three bronchioloaveolar carcinomas, two squamous cell carcinomas, one adenosquamous carcinoma, and one other type of NSCLC. No mutation was detected at codon 719 in exon 18 and codon 790 in codon 20. Seven tumors had in-frame deletions within exon 19, resulting in the loss of codons 746 through 750 in six tumors, and loss of codon 747 through 752 in one tumor. 12 cases had a 2573 T>G mutation resulted in L858R change. SNP -216 in the promoter region were detected in seven (8.6%) cases that include three adenocarcinomas, one bronchioloaveolar carcinoma, three squamous cell carcinomas. Three patients with SNP -216 in the promoter region had another mutation in exon 21 (L858R). Results of the multiplex PCR and primer extension mutation analysis of the *EGFR *gene are listed in Table 3.

**Table 3:** Mutation analysis of the *EGFR *gene by multiplex PCR and primer extension.

	**Case(s) with *EGFR *mutation(s)**				
	
**NSCLC subtype**		E746-A750 del		L747-S752 del	**L858R**
	**-216 G/T**	2235-2249 del	2236-2250 del	2240-2257 del	2573 T>G
Adenocarcinoma (n = 26)	3	1	3	0	8
Bronchioloalveolar carcinoma (n = 6)	1	2	0	0	1
Squamous cell carcinoma (n = 33)	3	0	0	0	2
Adenosquamous carcinoma (n = 5)	0	0	0	1	0
Others (n = 11)	0	0	0	0	1
**Total (n = 81)**	**7**	**3**	**3**	**1**	**12**

### Direct Sequencing

We also used direct sequencing to analyze the -216 promoter region and exons 18-21 of the *EGFR *gene. The results of sequencing analysis were identical to the results of multiplex PCR with primer extension analysis. No other mutation was identified by direct sequencing. We can therefore conclude that our method was very accurate in profiling the most common *EGFR *mutations in NSCLC.

## Discussion

EGFR proteins control essential signaling pathways that regulate cell proliferation [18]. Increased levels of *EGFR *gene expression are observed in many cancers, including NSCLC, and its expression is correlated with an adverse prognosis [2-4,19]. Clinical responsiveness to gefitinib and erlotinib in NSCLC have been shown to correlate with somatic mutations in the *EGFR *gene, which result in increased sensitivity to inhibition of growth by the drugs [8,10-12]. EGFR mutations have been found more frequently in non-smoking East Asian women with adenocarcinoma with bronchioalvelar features [14,20-26].

A protocol based on mutant-enriched PCR followed by primer extension of the PCR products was used to detect *EGFR *T790M mutation in NSCLC [27]. We recently demonstrated a simple and fast way to identify *K-RAS *mutation [17]. In this study, we extend the application to detect -216 promoter region and exons 18-21 mutations of *EGFR *gene simultaneously and apply this method to investigate the mutation status in 81 cases of NSCLC. We compared the results with that from direct sequencing for detecting *EGFR *mutations in 81 cases of NSCLC. The two protocols identified the same 26 mutations, but the new method is superior to direct sequencing in terms of the amount of work and time required. With this method, -216 promoter region and exons 18-21 of the EGFR gene were amplified with multiplex PCR in a single tube and the detection of mutations in the *EGFR *promoter and four key exons can be combined into one assay. This allows a sample to be screened for all common *EGFR *mutations simultaneously. We previously reported that that as little as 10 ng of DNA was enough for the multiplex PCR reaction and we also showed that this method can detect mutations against a background of up to at least 23 wild-type alleles [3]. Moreover, because the technique is a sequencing-based approach, additional sequencing is not necessary.

Distinguishing sequence variants with primer extension is based on the high accuracy of nucleotide incorporation catalyzed by a DNA polymerase. Current products of the thermostable enzymes used in primer extension have very low error rates and are specific for ddNTPs [28]. These characteristics provide negligible primer misincorporation and excellent discrimination between wild, heterozygous and homozygous genotypes. Another advantage of the primer extension reaction is its multiplexing capability, with several mutations being detected in a single reaction tube. Multiplex PCR-SSCP- or PCR-ARMS-based methods can also simultaneously detect several mutations. However, PCR-SSCP require further confirmation by direct sequencing, and PCR-ARMS require more primers than are possible in a single reaction to detect all mutations at -216 promoter region and exons 18-21 of the EGFR gene. The primer extension reaction is a less time-consuming assay because automated fluorescent capillary electrophoresis of the products requires only 25 minutes in comparison with capillary electrophoresis required for standard sequencing that takes more than an hour.

## Conclusions

The method that we demonstrated in this report provides a rapid way to identify common *EGFR *mutations for the purpose of clinical evaluation in NSCLC. The method can also be applied in the detection of other mutations in the *EGFR *gene.

## Competing interests

The authors declare that they have no competing interests.

## Authors' contributions

CHL performed PCR and primer extension and draft the manuscript, KTY participated in the design of the study, YSC performed direct sequencing, NCH participated in the analysis and helped to draft the manuscript, JGC designed the study.

## References

[B1] Brambilla E Travis WD Colby TV Corrin B Shimosato Y The new World Health Organization classification of lung tumours Eur Respir J 2001 18 6 1059 1068 10.1183/09031936.01.00275301 11829087

[B2] Hirsch FR Varella-Garcia M Bunn PA Jr Di Maria MV Veve R Bremmes RM Baron AE Zeng C Franklin WA Epidermal growth factor receptor in non-small-cell lung carcinomas: correlation between gene copy number and protein expression and impact on prognosis J Clin Oncol 2003 21 20 3798 3807 10.1200/JCO.2003.11.069 12953099

[B3] Nicholson RI Gee JM Harper ME EGFR and cancer prognosis Eur J Cancer 2001 37 Suppl 4 S9 15 10.1016/S0959-8049(01)00231-3 11597399

[B4] Ohsaki Y Tanno S Fujita Y Toyoshima E Fujiuchi S Nishigaki Y Ishida S Nagase A Miyokawa N Hirata S Kikuchi K Epidermal growth factor receptor expression correlates with poor prognosis in non-small cell lung cancer patients with p53 overexpression Oncol Rep 2000 7 3 603 607 10767376 10.3892/or.7.3.603

[B5] Veale D Ashcroft T Marsh C Gibson GJ Harris AL Epidermal growth factor receptors in non-small cell lung cancer Br J Cancer 1987 55 5 513 516 3038157 10.1038/bjc.1987.104 PMC2001730

[B6] Miller VA Kris MG Shah N Patel J Azzoli C Gomez J Krug LM Pao W Rizvi N Pizzo B Tyson L Venkatraman E Ben-Porat L Memoli N Zakowski M Rusch V Heelan RT Bronchioloalveolar pathologic subtype and smoking history predict sensitivity to gefitinib in advanced non-small-cell lung cancer J Clin Oncol 2004 22 6 1103 1109 10.1200/JCO.2004.08.158 15020612

[B7] Yang CH Shih JY Chen KC Yu CJ Yang TY Lin CP Su WP Gow CH Hsu C Chang GC Yang PC Survival outcome and predictors of gefitinib antitumor activity in East Asian chemonaive patients with advanced nonsmall cell lung cancer Cancer 2006 107 8 1873 1882 10.1002/cncr.22220 16989002

[B8] Byrne BJ Garst J Epidermal growth factor receptor inhibitors and their role in non-small-cell lung cancer Curr Oncol Rep 2005 7 4 241 247 10.1007/s11912-005-0045-6 15946581

[B9] Sequist LV Joshi VA Janne PA Bell DW Fidias P Lindeman NI Louis DN Lee JC Mark EJ Longtine J Verlander P Kucherlapati R Meyerson M Haber DA Johnson BE Lynch TJ Epidermal growth factor receptor mutation testing in the care of lung cancer patients Clin Cancer Res 2006 12 14 Pt 2 4403s 4408s 10.1158/1078-0432.CCR-06-0099 16857818

[B10] Pao W Miller V Zakowski M Doherty J Politi K Sarkaria I Singh B Heelan R Rusch V Fulton L Mardis E Kupfer D Wilson R Kris M Varmus H EGF receptor gene mutations are common in lung cancers from "never smokers" and are associated with sensitivity of tumors to gefitinib and erlotinib Proc Natl Acad Sci USA 2004 101 36 13306 13311 10.1073/pnas.0405220101 15329413 PMC516528

[B11] Paez JG Janne PA Lee JC Tracy S Greulich H Gabriel S Herman P Kaye FJ Lindeman N Boggon TJ Naoki K Sasaki H Fujii Y Eck MJ Sellers WR Johnson BE Meyerson M EGFR mutations in lung cancer: correlation with clinical response to gefitinib therapy Science 2004 304 5676 1497 1500 10.1126/science.1099314 15118125

[B12] Lynch TJ Bell DW Sordella R Gurubhagavatula S Okimoto RA Brannigan BW Harris PL Haserlat SM Supko JG Haluska FG Louis DN Christiani DC Settleman J Haber DA Activating mutations in the epidermal growth factor receptor underlying responsiveness of non-small-cell lung cancer to gefitinib N Engl J Med 2004 350 21 2129 2139 10.1056/NEJMoa040938 15118073

[B13] Kobayashi S Boggon TJ Dayaram T Janne PA Kocher O Meyerson M Johnson BE Eck MJ Tenen DG Halmos B EGFR mutation and resistance of non-small-cell lung cancer to gefitinib N Engl J Med 2005 352 8 786 792 10.1056/NEJMoa044238 15728811

[B14] Kosaka T Yatabe Y Endoh H Kuwano H Takahashi T Mitsudomi T Mutations of the epidermal growth factor receptor gene in lung cancer: biological and clinical implications Cancer Res 2004 64 24 8919 8923 10.1158/0008-5472.CAN-04-2818 15604253

[B15] Pao W Miller VA Politi KA Riely GJ Somwar R Zakowski MF Kris MG Varmus H Acquired resistance of lung adenocarcinomas to gefitinib or erlotinib is associated with a second mutation in the EGFR kinase domain PLoS Med 2005 2 3 e73 10.1371/journal.pmed.0020073 15737014 PMC549606

[B16] Liu W Innocenti F Wu MH Desai AA Dolan ME Cook EH Jr Ratain MJ A functional common polymorphism in a Sp1 recognition site of the epidermal growth factor receptor gene promoter Cancer Res 2005 65 1 46 53 15665278

[B17] Chang YS Yeh KT Chang TJ Chai C Lu HC Hsu NC Chang JG Fast simultaneous detection of K-RAS mutations in colorectal cancer BMC Cancer 2009 9 179 10.1186/1471-2407-9-179 19515263 PMC2702390

[B18] Oda K Matsuoka Y Funahashi A Kitano H A comprehensive pathway map of epidermal growth factor receptor signaling Mol Syst Biol 2005 1 2005 0010 10.1038/msb4100014 16729045 PMC1681468

[B19] Krause DS Van Etten RA Tyrosine kinases as targets for cancer therapy N Engl J Med 2005 353 2 172 187 10.1056/NEJMra044389 16014887

[B20] Janne PA Engelman JA Johnson BE Epidermal growth factor receptor mutations in non-small-cell lung cancer: implications for treatment and tumor biology J Clin Oncol 2005 23 14 3227 3234 10.1200/JCO.2005.09.985 15886310

[B21] Shigematsu H Lin L Takahashi T Nomura M Suzuki M Wistuba II Fong KM Lee H Toyooka S Shimizu N Fujisawa T Feng Z Roth JA Herz J Minna JD Gazdar AF Clinical and biological features associated with epidermal growth factor receptor gene mutations in lung cancers J Natl Cancer Inst 2005 97 5 339 346 15741570 10.1093/jnci/dji055

[B22] Han SW Kim TY Hwang PG Jeong S Kim J Choi IS Oh DY Kim JH Kim DW Chung DH Im SA Kim YT Lee JS Heo DS Bang YJ Kim NK Predictive and prognostic impact of epidermal growth factor receptor mutation in non-small-cell lung cancer patients treated with gefitinib J Clin Oncol 2005 23 11 2493 2501 10.1200/JCO.2005.01.388 15710947

[B23] Marchetti A Martella C Felicioni L Barassi F Salvatore S Chella A Camplese PP Iarussi T Mucilli F Mezzetti A Cuccurullo F Sacco R Buttitta F EGFR mutations in non-small-cell lung cancer: analysis of a large series of cases and development of a rapid and sensitive method for diagnostic screening with potential implications on pharmacologic treatment J Clin Oncol 2005 23 4 857 865 10.1200/JCO.2005.08.043 15681531

[B24] Mitsudomi T Kosaka T Endoh H Horio Y Hida T Mori S Hatooka S Shinoda M Takahashi T Yatabe Y Mutations of the epidermal growth factor receptor gene predict prolonged survival after gefitinib treatment in patients with non-small-cell lung cancer with postoperative recurrence J Clin Oncol 2005 23 11 2513 2520 10.1200/JCO.2005.00.992 15738541

[B25] Tsao MS Sakurada A Cutz JC Zhu CQ Kamel-Reid S Squire J Lorimer I Zhang T Liu N Daneshmand M Marrano P da Cunha Santos G Lagarde A Richardson F Seymour L Whitehead M Ding K Pater J Shepherd FA Erlotinib in lung cancer - molecular and clinical predictors of outcome N Engl J Med 2005 353 2 133 144 10.1056/NEJMoa050736 16014883

[B26] Sharma SV Bell DW Settleman J Haber DA Epidermal growth factor receptor mutations in lung cancer Nat Rev Cancer 2007 7 3 169 181 10.1038/nrc2088 17318210

[B27] Inukai M Toyooka S Ito S Asano H Ichihara S Soh J Suehisa H Ouchida M Aoe K Aoe M Kiura K Shimizu N Date H Presence of epidermal growth factor receptor gene T790M mutation as a minor clone in non-small cell lung cancer Cancer Res 2006 66 16 7854 7858 10.1158/0008-5472.CAN-06-1951 16912157

[B28] Syvanen AC From gels to chips: "minisequencing" primer extension for analysis of point mutations and single nucleotide polymorphisms Hum Mutat 1999 13 1 1 10 10.1002/(SICI)1098-1004(1999)13:1<1::AID-HUMU1>3.0.CO;2-I 9888384

